# Complete mitochondrial genome of the Northern Long-eared Owl (*Asio otus* Linnaeus, 1758) determined using next-generation sequencing

**DOI:** 10.1080/23802359.2018.1451260

**Published:** 2018-04-23

**Authors:** Mu-Yeong Lee, Seon-Mi Lee, Hey Sook Jeon, Sang-Hwa Lee, Joon-Young Park, Junghwa An

**Affiliations:** a DNA Analysis Division, Seoul Institute, National Forensic Service, Seoul, Republic of Korea;; b BK21 PLUS Program for Creative Veterinary Science Research and Conservation Genome Resource Bank for Korean Wildlife (CGRB), College of Veterinary Medicine, Seoul National University, Seoul, Republic of Korea;; c Animal Resources Division, National Institute of Biological Resources, Incheon, Republic of Korea;; d Graduate Program in Cellular Biology and Genetics, College of Medicine, Chungbuk National University, Cheongju, Republic of Korea;; e Department of Veterinary Medicine, National Institute of Ecology, Chungnam, Republic of Korea

**Keywords:** *Asio otus*, mitochondrial genome, Long-eared Owl, next generation sequencing

## Abstract

In this study, the mitogenome of *Asio otus*, the Northern Long-eared Owl, was analysed using Illumina next-generation sequencing. The mitogenome was found to be a circular molecule, 17,735 bp long with a slight AT bias (53.0%). The gene arrangement pattern was the same as that of a typical vertebrate, containing 37 genes (13 protein-coding genes (PCGs), 22 transfer RNA genes, two ribosomal RNA genes, and a non-coding control region). In the putative control region (1984 bp), there were two types of tandem repeats at the end of the region. A phylogenetic tree was constructed using the 13 PCG sequences discovered in this study and those of that have been previously published of other Strigidae species and revealed a close relationship between *A*. *otus* and *A. flammeus.* The newly generated mitogenome from this study enriches the genomic resources available for future evolutionary studies and promotes conservation genetics of this species.

The Northern Long-eared Owl, *Asio otus* (Linnaeus, 1758), is a medium-sized, nocturnal bird and has a large, round head, and noticeable ear tufts. It is widely distributed in Holarctic region, its range extending throughout temperate North America and in Europe, Russia, and Japan. In addition, isolated populations in northern and eastern Africa, the Azores, and the Canary Island have been reported (Marks et al. 1994; Olsen and Marks 2018). The species population has been declining due to habitat loss and the expansion of competing species. The species is classified as ‘Least Concern’ on the IUCN Red List of Threatened Species. In South Korea, this species is distributed throughout the region, but it is rare. Currently, the Northern Long-eared Owl is classified as a Natural Monument (No. 324-5) by the Cultural Heritage Administration of Korea. In this study, we characterized the complete mitogenome sequence of *A. otus* using Illumina next-generation sequencing technology and performed an analysis of the phylogenetic relationships among Strigidae species with the available data in GenBank.

For this study, a specimen (IN830) was collected from Dang-jin, Chungchungnam-do, South Korea. Whole genomic DNA was extracted from the specimen’s blood using a DNeasy^®^ Blood & Tissue Kit (Qiagen, Valencia, CA) in accordance with manufacturer’s protocol. Next-generation sequencing was performed using an Illumina HiSeq 2500 platform at the NICEM (National Instrumentation Center for Environmental Management), Seoul, South Korea. DNA sequences were assembled and edited with the software Geneious Pro v10.2.3 (Biomatters Ltd, Auckland, New Zealand; Kearse et al. 2012). All protein-coding and two ribosomal RNA genes were annotated using the online tool DOGMA (Wyman et al. 2004) and software ARWEN (Laslett and Canbäck 2008).

The mitogenome sequence of *A. otus* was 17,735-bp long and was submitted to GenBank under the accession number MG916810. The gene arrangement of *A. otus* was largely the same as that of previously reported mitogenomes for other Strigidae species (Zhang et al. 2015) and consisted of 13 protein-coding genes (PCGs), 22 transfer RNA genes (tRNAs), two ribosomal RNA genes (rRNAs), and a putative control region (D-loop). Except for the *ND6* gene and eight tRNA genes that are encoded by the L-strand, the remaining genes were encoded by the H-strand. The overall nucleotide composition was 30.1% A, 33.5% C, 13.5% G, and 22.9% T with a slight AT bias (53.0%). In the *ND3* gene, a frameshift mutation was detected at the 174th bp, which is also found in *Asio flammeus* (KP889214). In the putative control region (1984 bp in length), located between *tRNA-Phe* and tRNA*-Glu*, two tandem repeats were identified at positions 11,465–11,845 bp and 11,846–12,138 bp.

To reveal the phylogenetic relationship of *A. otus* within the Strigidae, a phylogenetic tree was constructed based on the concatenated identified 13 PCGs using the neighbour-joining (NJ) method as implemented in MEGA v7.0 (Tamura et al. 2013). The *Phodilus badius* (in the family Tytonidae) mitogenome (accession number: NC023787) was used as an outgroup for tree rooting. The NJ tree showed two clades; *A. otus* formed a group with species in the genera *Strix*, *Otus*, *Ninox*, and *Asio*, while species in the genus *Glaucidium* belonged to the other clade. In addition, *A. otus* was placed closest to the species from the same genus, *A*. *flammeus,* with a high bootstrap value (Figure 1). The newly generated mitogenome in this study enriches the genomic resources available for further evolutionary studies and can play a significant role in conservation genetics.

**Figure 1.**
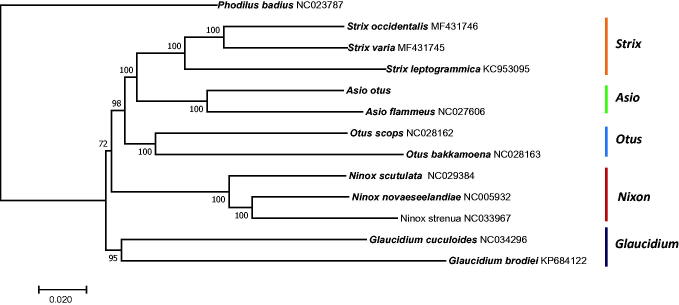
Neighbour-joining tree of 12 species of Strigidae based on the concatenated nucleotide sequences of the 13 protein-coding genes from the mitogenomes of each species. Bootstrap values are shown at the nodes. GenBank accession numbers for the sequences are indicated next to species designations.
